# Impact of experience on visual behavior and driving performance of high-speed train drivers

**DOI:** 10.1038/s41598-022-10055-9

**Published:** 2022-04-08

**Authors:** Yang Du, Jin-yi Zhi, Si-jun He

**Affiliations:** School of Design, Southwest Jiaotong University, Chengdu, 611756 China

**Keywords:** Human behaviour, Operant learning, Pattern vision

## Abstract

This study investigated the impact of experience on the visual behavior and driving performance of high-speed train drivers, and explored the correlation between visual behavior and driving performance. Through a simulated driving task, eye movement data and operating data of novice drivers, trainee drivers, and experienced drivers in the traction stage, normal operation process stage, and braking stage were collected. Variance and linear regression were used to analyze the difference and correlation between indicators. The results show that experience could change the driver’s information collection method from long fixation to multi-frequency. Experience also increased the consistency of group operations and reduced the likelihood of hazard occurrences. Therefore, driving performance can be improved by reducing the average fixation duration of information through interface optimization.

## Introduction

Train drivers, occupying a front-line position in the rail traffic sector, play a significant role in the safe operation of trains. Driving performance is an indicator of the operational capability of drivers. The Electric Multiple Units (EMUs) operating procedures require that when drivers control a train, they should keep their speed close to the authorized speed and run the train smoothly to ensure safe, on-time arrival. On-time percentage (PERCENT) is the most intuitive indicator to reflect the driving performance. Overspeed magnitude (MEAN) and overspeed time (SUM) are also important factors that affect driving safety, as exceeding the authorized speed may cause dangerous driving events, and the longer the overspeed time, the greater the danger. Studies have shown that experience plays a moderating role in safety performance^1^. The more experienced a driver is, the greater the speed perception and operational stability they have, the fewer speeding violations and less dangerous driving behavior they have, and the fewer accidents or near-accidents they have^1,2^. Experienced drivers can detect imminent danger in time and take initiatives to prevent dangerous situations^3^. Novice drivers react more strongly to danger, especially young drivers, who underestimate the risks and overestimate their driving skills. For example, the accident rate of young male drivers is higher than that of older male drivers, who have driven for a much longer time^4^.

The influence of driver cognition on driving performance has received extensive attention, as cognition is an important factor in ensuring driving safety^5^. Research in the automotive field has focused on general workload, visuospatial skills, attention, distraction, executive function, memory, and psychomotor skills^6–8^. High-speed train driving is a highly visual task owing to the one-way track running characteristics and high repeatability of driving action, and the cab interface provides comprehensive train operation information and route information to drivers. Drivers’ attention, memory, and visual perception impact driving performance^1^. There are three typical working stages of the train driving process: the traction stage, where the driver speeds up the train and reaches the authorized speed; the normal operation process stage, where the driver drives the train at the authorized speed; and the braking stage, where the driver decelerates and stops the train^9^. In all of these stages, the driver needs to search, locate, identify, and process all the information related to the position and state of the train and perform accordingly. Drivers’ attention can positively predict safety performance, while missed observations of line signals, speed limit signs, or alarms are common antecedents of risky behavior^10^.

Eye movement is a typical feature of visual behavior. In the information search process, saccades, which are rapid movements of the eye as it moves from one point of fixation to another, can realize the rapid search of the view field and selected stimulus information. Fixation can provide necessary visual information to drivers, including train and environmental information. Eye movement indicators can reflect the way of obtaining information, the number of fixations reflects the drivers’ fixation target and area of interest, the fixation duration reflects the difficulty in extracting valid information, and saccade amplitude reflects the amount of information captured in fixations. Experience has been found to cultivate driver information perception skills and strategies, thus leading to a more effective and functional visual faculty^11^. Underwood^12^ posited that the change in visual search strategy marks a transition from an inexperienced to an experienced driver. Novice drivers have been found to have a longer processing time and a narrower lateral search range than experienced drivers^13^. Guo et al.^14^ pointed out that at low speeds, the fixation frequency of experienced drivers on nearby targets was 18% higher than that of inexperienced drivers, while at high speeds, the fixation frequency of experienced drivers on distant areas was approximately 2.4 times that of new drivers. In demanding sections, experienced drivers have been found to be more sensitive to the complexity of the road than novice drivers, and, as scenarios evolve, they pay more attention to visual information^15–17^. When a vehicle runs in a narrow horizontal place, skilled drivers have been found to increase the fixation frequency on the speedometer, while newcomers notice obstacles more, indicating that new drivers tend to shift their attention to threatening stimuli, while practiced drivers are more likely to consciously monitor task goals^18^. Ma et al.^19^ found that experienced drivers responded faster under dangerous circumstances, and experience could help improve drivers’ processing efficiency of danger information. Novice drivers have also been found to have a longer fixation duration than experienced drivers in dangerous driving conditions, thereby reducing the probability of discovering danger, as such conditions are detrimental to safe driving^20,21^. One explanation for this phenomenon is that novice drivers are used to continuing their usual fixation patterns while driving^15^. Other studies in transportation have also reflected the impact of experience on visual behavior and driving performance. Bazargan and Guzhva^22^ found that experienced pilots had better visual perception and reactions in complex tasks than inexperienced pilots.

In recent years, against the backdrop of rapidly developing high-speed rails in the world, the demand for drivers has grown with increased high-speed Electric Multiple Units (EMUs). Efficiently training many train drivers has become a common concern of railways. Owing to the low utilization of equipment, high cost, and risk of accidents during training in a real vehicle, simulators have become the preferred method for training^23^. Although studies in other fields have shown that experience affects driving performance, there has been little research on high-speed rail driving to define and quantify this relationship. The purpose of this study was to clarify the impact of experience on drivers’ visual behavior and driving performance as well as the correlation between visual behavior and driving performance. The research results provide empirical assistance for cab display interface optimization and driver training.

## Methods

### Participants

This study recruited 14 students majoring in rail vehicles as novice drivers (group A), 14 high-speed trainee drivers who had a license for 1 year (group B), and 14 high-speed drivers with more than 3 years driving experience (group C). Participants were all male, aged 23 to 33 years (M = 27.07, SD = 3.11), righthanded, with no known dyskinesia or sleep problems, and with normal vision or corrected vision.

This study was approved by the Ethics Committee of Southwest Jiaotong University and conducted according to the principles of the Declaration of Helsinki. All the participants provided written informed consent before participating.

### Apparatus

Simulators are often used to study the influence of individual differences and external factors on driving performance^24^. They exclude the risk of accidents or injuries that may occur in real operation, and offer the possibility of finding problems in a standardized scenario at a low cost and with high efficiency^25^. Gotardi et al.^26^, Niu et al.^27^, Underwood et al.^28^, Brandtner et al.^29^ also used simulators to replace real scenes and demonstrated that this simplified experimental setup was effective.

**Figure 1:**
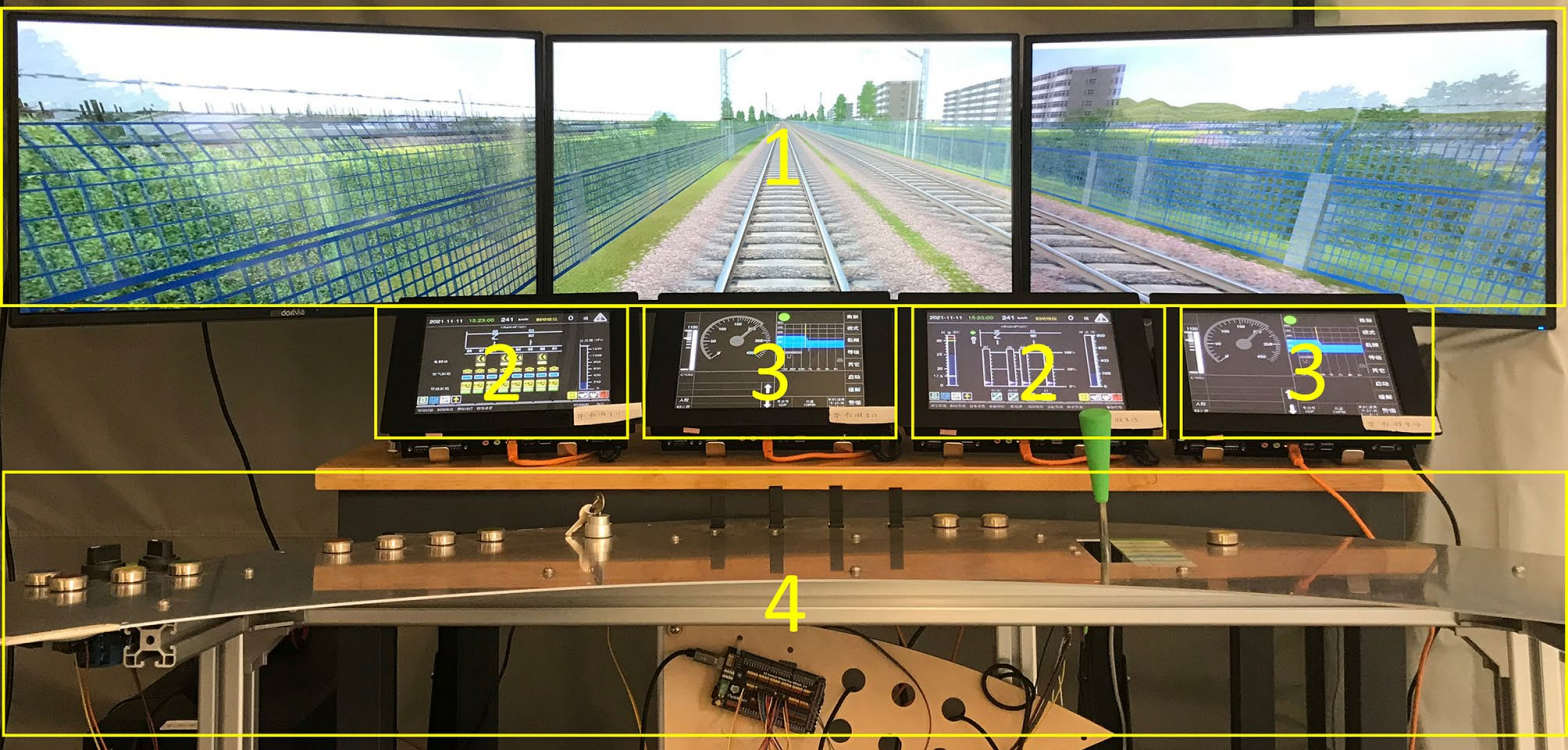
Simulated driving device. The simulation scenarios are displayed on Area 1. Area 2 is the Train Control and Monitor System (TCMS) display screen, which contains information about the vehicle’s state. Area 3 is the Automatic Train Protection (ATP) display screen, which contains information about train driving and protection. The simulator console is located in Area 4.

**Figure 2:**
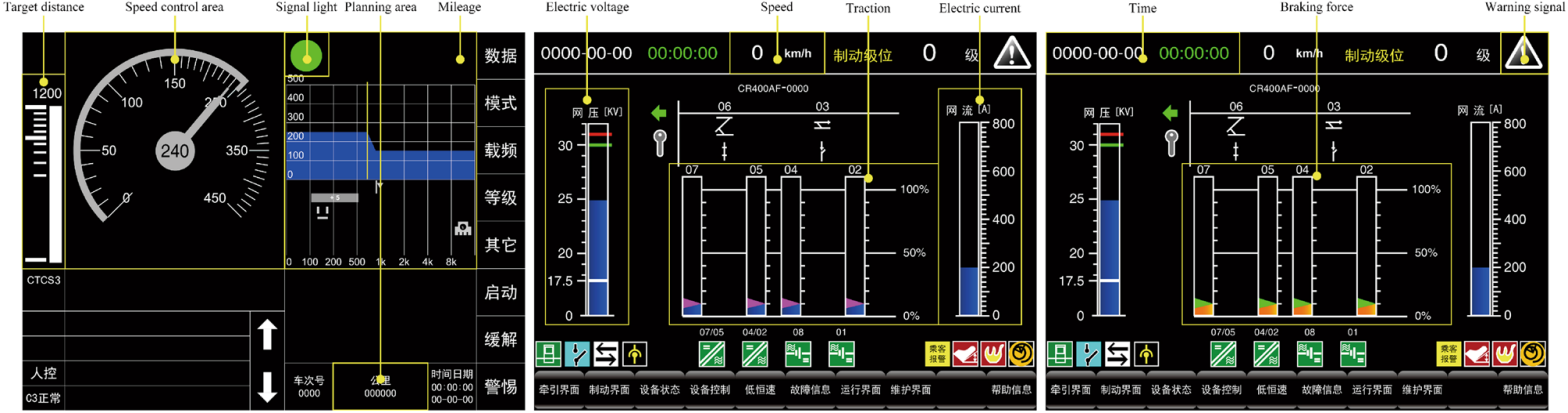
An example of the ATP display (left), the TCMS display of traction status (middle) and the TCMS display of braking status (right).

In this study, a simulated driving device was designed with reference to the cab console of China's CR400AF high-speed EMU, featuring simulation scenarios created with the open-source software Open Rails and data linkage realized with Java software. Scenes outside the vehicle were displayed with a computer and three 24-inch screens, with actual route data and train operation data shown on four 10.4-inch touch-screen industrial tablets. The layout of the display screen, console, and driver's seat of the simulator was similar to that of the CR400AF EMU. The environmental brightness in the experiment was about 150 lx, which met the illumination requirements of the CR400AF EMU cab. During the experiment, a Tobii Pro Glasses 2 eye tracker was used to record the eye movement data of the subjects. The simulated driving device set up in this study is shown in Fig. 1, and the interface information is shown in Fig. 2.

### Experiment design

The simulated driving experiment selected the Beijing–Tianjin intercity railway as the route. This route is a standard driving route, starting from Beijing South Railway Station and ending at Tianjin Station, a route which is 120 km long and has a maximum operating speed of 350 km/h. The running time is 30 min, excluding intermediate stops, which meets the experimental requirements and reduces the impact of fatigue. The experiment consisted of three steps: interpretation and demonstration, familiarity with the process, and the formal experiment. First, participants learned about the tasks and operating rules and observed a driving demonstration by the researchers. Then, they simulated driving training for no less than 2 h under the guidance of the researchers. Subsequently, they were all able to complete the driving task without any help. During the formal experiment, participants were required to wear a Tobii eye tracker to complete different simulated driving stages and were allowed to correct inappropriate actions countless times during driving. When the experiment had finished with data properly recorded, participants were asked to complete the Situation Awareness Rating Technique (SART) and NASA Task Load Index (NASA-TLX) questionnaires.

This study adopted a 3 × 3 design; the independent variable within the participant group was the driving stage (traction, normal operation process, and braking), and the independent variable between the groups was driving experience (groups A, B, and C). Driving performance (on-time percentage, overspeed magnitude, and overspeed time), visual behaviors (saccade amplitude, number of fixations, average fixation duration), and subjective ratings (situational awareness and task load index) were used as dependent variables.

### Data processing

Researchers adopted descriptive statistics for the dependent variable results. The Shapiro–Wilk method was used for checking the normal distribution of all dependent variables; analysis of variance (ANOVA) was used for checking the variance homogeneity and differences between groups; and linear regression was used to analyze the correlations between dependent variables. All statistical analyses were carried out in SPSS 24 software.

## Results

### Driving performance

Table 1 lists the on-time percentage, overspeed magnitude, and overspeed time of the three driving stages. The on-time percentage of Group A was 86.2%, the overspeed magnitude was 13.11%, and the overspeed time was 173.4 s. For the on-time percentage, group B had a promotion of 10% more than group A; group C had a promotion of 4% more than group B and 15% more than group A. For the overspeed magnitude, group B had a decrease of 58% more than group A; group C had an decrease of 79% more than group B and 91% more than group A. For the overspeed time, group B had a decrease of 38% more than group A; group C had a decrease of 94% more than group B and 96% more than group A. Drivers’ driving performance improved significantly as their experience grew.

**Table 1:** Impact of driving experience on driving performance.

Variables	Participants			ANOVA	
	Group A	Group B	Group C	F-value	*p*-value
On-time percentage	86.163	94.698	98.825	22.250	< 0.001
Overspeed magnitude	13.111	5.492	1.130	23.010	< 0.001
Traction stage	16.285	6.825	0.573	7.757	0.001
Normal operation process stage	6.792	1.041	0.000	46.404	< 0.001
Braking stage	14.999	8.592	1.277	7.346	0.002
Overspeed time	173.360	106.640	6.07	13.852	< 0.001
Traction stage	32.500	24.000	2.00	46.223	< 0.001
Normal operation process stage	75.000	34.640	0.00	29.481	< 0.001
Braking stage	65.860	48.000	4.07	102.774	< 0.001

**Figure 3:**
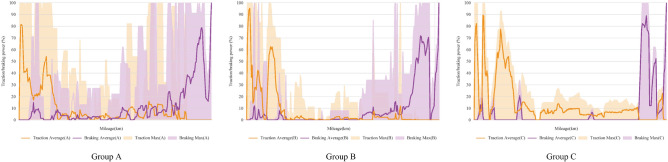
Driving operations of the three groups. The operating differences between participants were considerable. However, the difference between the groups could not be clearly shown using the average ± standard deviation, so the average value and the maximum value were used to present the traction and braking power. The minimum value was 0, which is not shown in the figure.

**Figure 4:**
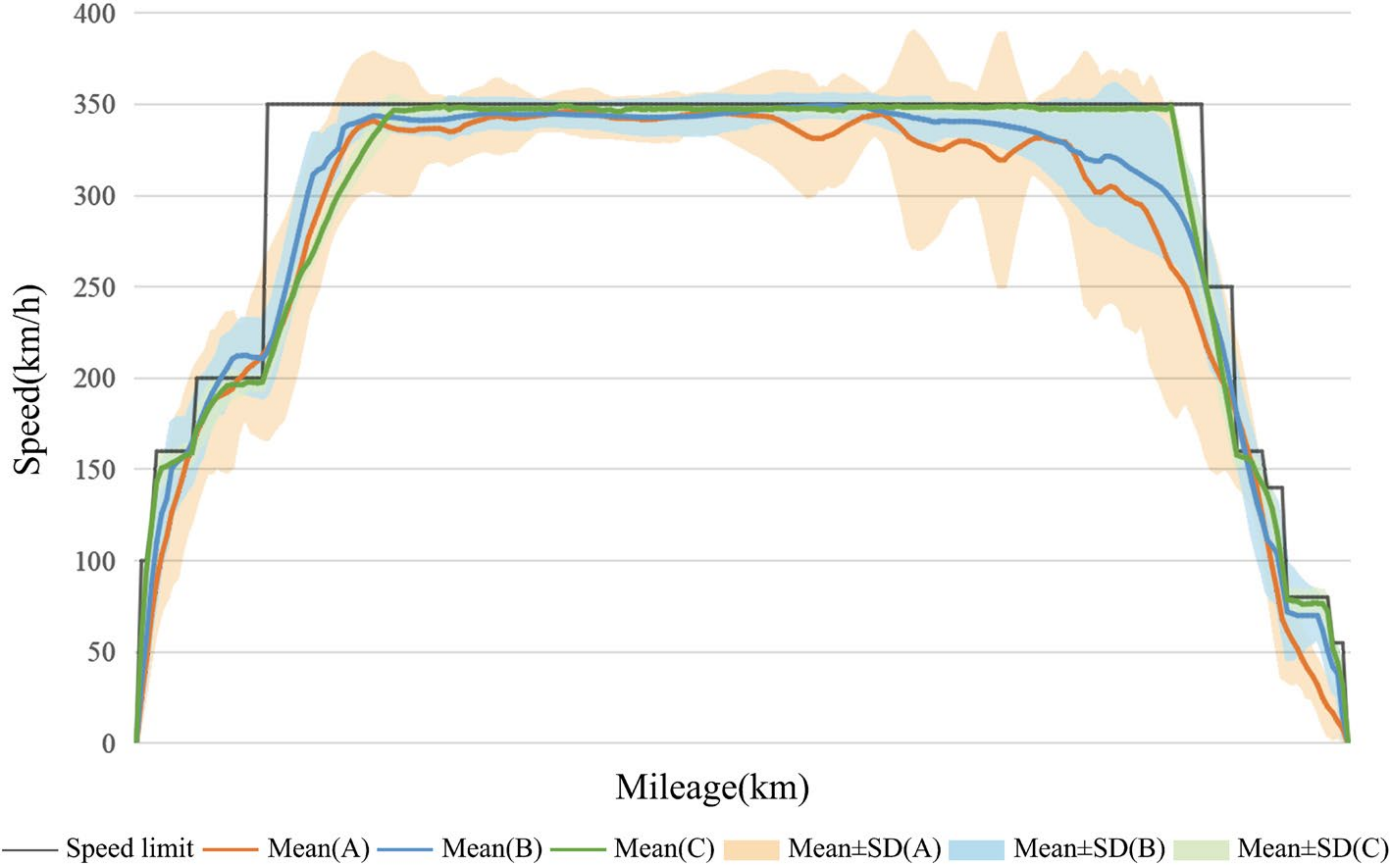
The train speed curve of groups A, B, and C.

The main operations of the participants throughout the driving process were the application, adjustment, and cancellation of traction and braking. Figure 3 illustrates the actions of the three groups. Group C performed more consistently, group A performed in a more dispersed manner, and group B was at an intermediate level between groups A and C. In the traction stage, the traction operation of group A lagged behind that of group B and C, and was the maximum traction power applied in Group B. In the normal operation process stage, 57% of participants in group A performed repeated braking-traction-braking alternations, group A increased its braking power halfway through the normal operation process stage, group B braked with a 10-km delay after group A and 30 km ahead of group C. Figure 4 illustrates the train speed changes of the three groups, showing that the change trends were consistent with the application of traction and braking. The train speed curve of group C was closest to the authorized speed, the speed curve of group B was more consistent than that of group A, and the fluctuation within the group was also smaller.

### Visual information

Group B and C paid had one more fixation on information per minute than group A, but reduced about 0.1 s in the average fixation duration compared with group A, and the saccade amplitude was increased by 1 degree compared with group A (Table 2). That is, experience could improve the drivers’ attention to information, speed up the extraction efficiency from information, and increase the information captured amount per fixation.

**Table 2:** Impact of driving experience on visual information.

Variables	Participants			F-value	*p*-value
	Group A	Group B	Group C		
**Number of fixations**					
Traction stage	1.756	3.427	3.139	6.859	0.003
Normal operation process stage	1.796	2.395	2.494	4.456	0.018
Braking stage	1.377	2.792	3.462	11.542	< 0.001
**Average fixation duration**					
Traction stage	0.445	0.413	0.350	5.976	0.005
Normal operation process stage	0.471	0.309	0.327	3.506	0.040
Braking stage	0.445	0.295	0.369	7.146	0.002
**Saccade amplitude**					
Traction stage	6.012	6.872	7.456	4.818	0.013
Normal operation process stage	5.974	6.471	7.282	5.581	0.007
Braking stage	5.976	6.253	6.481	0.957	0.393

Eye movement data of the participants are described in Table 3. In the traction stage, the three groups had one or more fixations per minute on the target distance, speed control area, planning area, current speed, traction, and braking force, indicating that this information was the focus at this stage. The fixation number of group B and C for the speed control area, current speed, and traction was higher than that of group A, while the fixation number of group B for braking force was higher than that of groups A and C. The average fixation duration of group A for the target distance, speed control area, traction, and braking force was 0.2–0.5 s longer than that of groups B and C. The average fixation duration of group B for the target distance and planning area was 0.1 s longer than that of group C. In the normal operation process stage, the three groups had one or more fixations per minute on the target distance, speed control area, mileage, planning area, current speed, and traction. The fixation number of groups B and C for the planning area and current speed was higher than that of group A, while the traction and braking force was lower than those of group A. The average fixation duration of group A for the traction and braking force was 0.2–0.4 s longer than that of groups B and C. In the braking stage, the three groups had one or more fixations per minute on the speed control area, planning area, current speed, traction, and braking force. The fixation number of groups B and C for the speed control area, planning area, current speed, and braking force was higher than that of groups A, while the traction for group C was lower than that of group A and B. The average fixation duration of group A for the speed control area, current speed, and braking force was 0.2–0.7 s longer than that of groups B and C, and group B’s speed control area and current speed were 0.1–0.2 shorter than those of group C.

**Table 3:** Number of fixations and average fixation duration of the three groups on the interface information.

Stage	Number of fixations											
	Traction				Normal operation process				Braking			
Information	A	B	C	*p*	A	B	C	*p*	A	B	C	*p*
Target distance	1.85	2.32	2.13	0.446	2.52	2.24	2.65	0.838	0.46	2.07	2.34	0.001
Speed control area	1.77	9.07	8.88	< 0.001	2.31	4.62	4.96	0.066	1.12	5.82	9.32	< 0.001
Locomotive signal	0.04	0.32	0.57	0.001	0.03	0.21	0.25	0.042	0.27	0.07	0.35	0.072
Mileage	0.77	0.79	0.77	0.993	1.87	1.74	1.69	0.954	0.31	0.04	0.72	0.003
Planning area	9.19	10.54	10.57	0.595	3.68	8.15	8.47	< 0.001	6.84	10.11	10.83	0.021
Electric voltage	0.08	0.25	0.37	0.024	0.06	0.57	0.49	0.004	0.29	0.15	0.27	0.142
Date and time	0.15	0.29	0.18	0.609	0.08	0.89	0.63	0.021	0.00	0.21	0.20	0.078
Current speed	0.85	4.61	5.77	< 0.001	2.08	6.77	6.98	0.010	1.35	3.39	5.22	0.006
Traction	1.81	5.18	4.73	< 0.001	3.76	0.96	1.52	< 0.001	2.07	3.00	1.45	0.081
Braking force	2.77	6.18	1.23	< 0.001	4.99	0.63	0.82	< 0.001	2.27	7.82	8.67	0.002
Electric current	0.23	1.43	1.45	0.001	0.14	0.87	0.12	< 0.001	0.57	0.31	0.98	0.005
Warning signal	1.57	0.15	1.01	<0.001	0.06	1.09	1.34	0.155	1.00	0.50	1.19	0.619

Stage	Average fixation duration											
	Traction				Normal operation process				Braking			
Information	A	B	C	*p*	A	B	C	*p*	A	B	C	*p*
Target distance	0.73	0.54	0.42	0.029	0.74	0.59	0.48	0.305	0.60	0.35	0.50	0.058
Speed control area	0.94	0.46	0.50	< 0.001	0.66	0.51	0.46	0.051	1.05	0.31	0.42	< 0.001
Locomotive signal	0.08	0.22	0.14	0.344	0.17	0.04	0.22	0.086	0.23	0.03	0.20	0.122
Mileage	0.31	0.50	0.47	0.029	0.79	0.54	0.53	0.282	0.94	0.82	0.82	0.291
Planning area	1.00	0.99	0.86	0.368	0.65	0.51	0.59	0.074	0.11	0.45	0.44	0.157
Electric voltage	0.05	0.21	0.12	0.097	0.38	0.07	0.13	0.095	0.22	0.24	0.21	0.501
Date and time	0.07	0.17	0.14	0.329	0.11	0.29	0.20	0.116	0.00	0.11	0.12	0.082
Current speed	0.33	0.44	0.42	0.060	0.58	0.17	0.32	0.021	0.60	0.16	0.38	0.003
Traction	0.90	0.46	0.46	0.002	0.60	0.54	0.45	0.666	0.49	0.38	0.47	0.359
Braking force	0.68	0.31	0.32	0.016	0.48	0.17	0.21	0.018	0.65	0.42	0.44	0.004
Electric current	0.17	0.46	0.24	0.030	0.48	0.12	0.17	0.080	0.33	0.10	0.24	0.123
Warning signal	0.08	0.20	0.11	0.112	0.01	0.17	0.15	0.051	0.14	0.17	0.19	0.365

### Subjective ratings

The level of situational awareness of groups B and C differed little. The demand for resources was about 40–45% lower than that of group A, the understanding of the situation was about 70–78% higher than that of group A, and the supply of resources was 56–67% higher. The mental demands of group C to complete the task were 16% lower than that of group B, while those of group B were 26% lower than that of group A. Groups B and C did not differ much in the temporal demand and efforts; these were about 43% and 29% lower, respectively, than those for group A. That is, the situational awareness, temporal demand, and efforts of experienced and inexperienced drivers were considerably different. With the increase of the driving age, the level of situational awareness, temporal demand, and efforts did not change significantly, but it could reduce the drivers’ mental demand.

### Correlation analysis

In order to research the correlation between driving performance and visual behavior, the driving performance indicators were used as the dependent variable, the eye movement indicators were used as the independent variable for linear regression analysis, screening and removal criteria were multicollinearity (VIF > 2) and significance effects (p > 0.05), and the following regression equation was obtained:

1 $$\mathrm{On\,time\,percentage}=68.125+1.872\times {\mathrm{NF}}_{\mathrm{P}}+\mathrm{Saccade\,amplitude}-{\mathrm{AFD}}_{\mathrm{S}}-{\mathrm{AFD}}_{\mathrm{B}}$$

2 $$\mathrm{Overspeed\,magnitude}=14.253-1.883\times {\mathrm{NF}}_{\mathrm{P}}-1.134\times {\mathrm{NF}}_{\mathrm{C}}-9.275\times {\mathrm{NF}}_{\mathrm{L}}+1.124\times {\mathrm{NF}}_{\mathrm{B}}+18.796\times {\mathrm{AFD}}_{\mathrm{S}}+23.051\times {\mathrm{AFD}}_{\mathrm{E}}$$

3 $$\mathrm{Overspeed\,time}=102.523-18.688\times {\mathrm{NF}}_{\mathrm{S}}-213.189\times {\mathrm{AFD}}_{\mathrm{C}}+124.741\times {\mathrm{AFD}}_{\mathrm{P}}+119.282\times {\mathrm{AFD}}_{\mathrm{S}}$$

NF_P_ in Eq. (1) represents the planning area fixation numbers, similar to NF_P_, NF_C_, NF_L_, NF_B_, and NF_S_, which represent the current speed, locomotive signal, braking force, speed control area, respectively. AFD_P_ in Eq. (3) represents the planning area and average fixation duration, similar to AFD_P_, AFD_S_, AFD_B_, AFD_E_, and AFD_C_, which represent the speed control area, braking force, electric voltage, current speed, respectively.

The adjusted R^2^ = 0.803 of the on-time percentage fitting equation, the adjusted R^2^ = 0.798 of the overspeed magnitude fitting equation, and the adjusted R^2^ = 0.702 of the overspeed time fitting equation. The three regression equations were well fitted, and all the independent variables in the equation had a significant effect on the dependent variable.

In summary, increasing the saccade amplitude and fixation numbers of the planning area, current speed, locomotive signal, and speed control area; increasing the average fixation duration of the current speed; and reducing the fixation numbers of the braking force and average fixation duration of the speed control area, braking force, electric voltage, and planning area could effectively improve driving performance.

**Figure 5:**
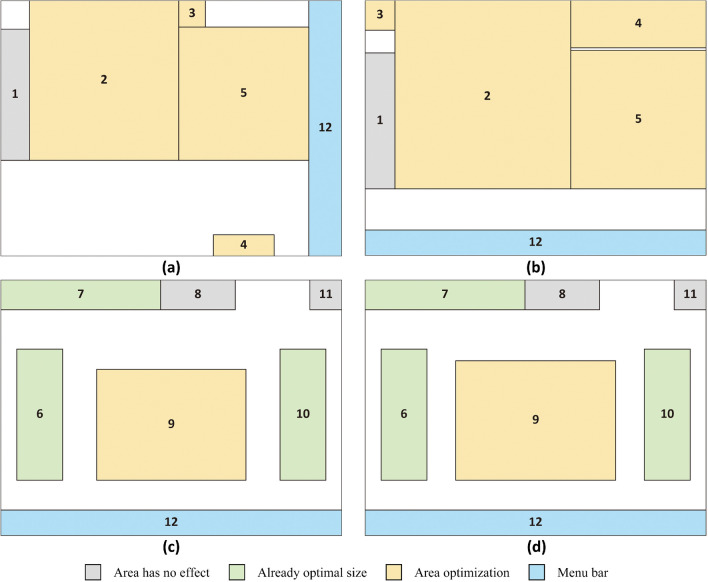
Layout optimization of ATP and TCMS display. (**a**) represents the original layout of ATP, (**b**) represents the layout of ATP after optimization. (**c**) represents the original layout of TCMS, (**d**) represents the layout of TCMS after optimization. 1 is the Target distance, 2 is the Speed control area, 3 is the Locomotive signal, 4 is the Mileage, 5 is the Planning area, 6 is the Electric voltage, 7 is the Data and time, 8 is the Current speed, 9 is the Traction/Braking force, 10 is the Electric current, 11 is the Warning signal, 12 is the Menu bar.

The saccade amplitude and fixation numbers rising gradually with driving experience increased, the average fixation duration could be decreased by changing the information area. Researchers compared the information extraction efficiency of different areas of the current speed, speed control area, braking force, electric voltage, and planning area, and found the range with faster extraction. The original area of the current speed was 35 × 14 mm (width × height, the same below), and the area had no significant effect on it. The original area of speed control area was 70 × 75 mm, and when the range was between 82 × 88 mm to 116 × 124 mm, the information extraction speed increased by 0.01–0.07 s. The original area of braking force was 70 × 52 mm, and when the range was larger than 75 × 56 mm, the information extraction speed increased by 0.1 s. The original area of electric voltage was 21.5 × 61.5 mm, and when the range was larger than 17.5 × 50 mm, there was no obvious difference in the extraction speed. The original area of the planning area was 61 × 62.5 mm, when the range was larger than 63.5 × 65 mm, the information extraction speed increased by 0.05 s. Researchers also experimented with the extraction efficiency of other information and optimized the original interface layout (Fig. 5).

## Discussion

Experience could improve driving performance, improve the on-time percentage, and reduce the likelihood of hazard occurrences. The average delay time of novice drivers was 249 s, which is 154 s longer than that of trainee drivers and 228 s longer than that of experienced drivers. This indicates that novice drivers need more time to complete driving. The average overspeed magnitude of trainee and experienced drivers was less than 10%, and the average overspeed of novice drivers was greater than 10%. Referring to high-speed railway operating regulations, when the speed exceeded 10% of the speed limit range, the probability of hazard occurrences was greatly increased. This indicates that after novice drivers become trainee drivers after professional training, the accident probability would drop by 38%, and after 3 years of vehicle operation, the probability of accidents would drop by 94%.

The on-time percentage of half of the novice drivers was equal to that of trainee drivers, with a short training period, and some of the beginners performed almost as well as the proficient drivers. However, the greatest difference between them and experienced drivers was in the consistency of group operation. That is, a longer driving experience decreased drivers’ individual performance differences, and even though they may have come from different locomotive depots, they were able to budget their time better and knew the machine more thoroughly. This agrees with Xu’s finding that drivers with increased driving experience were more sensitive to situational cues than personal factors, and thus were more likely to act as their peers^30^. In addition to the difference in situational perception between novice and experienced drivers, one explanation for the novices’ worse driving performance is that although they may have quickly mastered the necessary skills to control the vehicle, it takes longer to develop a standard operation process and expert driving skills^31^.

Experience could transform the visual behavior characteristics of drivers to form more targeted information search patterns. By comparing the visual behaviors in three driving stages, it was demonstrated that trainee and experienced drivers obtained information through frequent but short fixations, and the saccades were conducive to their broader observation of the whole scene and the overall control of information. Novices, however, obtained information by less frequent but longer gazes, which reduced their ability to identify danger. It was also revealed in the study of Underwood et al.^15,16^ that novice drivers had limited remaining focus owing to their devotion of most attentional resources to vehicle control. Therefore, they focused their search for information in smaller areas with narrower scans, thus discovering dangers more slowly than skilled drivers^32–34^. In addition, the level of situational awareness of the novice drivers was worse than that of experienced drivers. Dehais et al.^35^ and Peißl et al.^36^ proposed that the precursor to the lack of situational awareness manifests as increased visual attention to specific information, while drivers’ mental demand increases, and visual search behavior decreases. This is consistent with the experimental conclusions of this paper. The focus of trainee and experienced drivers in the three driving stages changed. In the traction and braking stage, they paid more attention to the speed control area and planning area than they in the normal operation process stage and paid less attention to the mileage and current speed than in the normal operation process stage. The difference between trainee drivers and experienced drivers was that in the traction and braking force, experienced drivers paid more attention to the traction in the traction stage and braking force in the braking stage, while trainee drivers paid more attention to both the traction and braking force. The information focus of novice drivers did not change significantly with the driving stage. Aksan et al.^5^ posited that experienced drivers have a procedural understanding of the task, so they can search for information purposefully and quickly switch between different information while driving.

Driving performance was positively correlated with the fixation number and negatively correlated with the fixation duration. Interface optimization could be adopted to narrow the gap caused by experience. One reason for the worse performance of novices is that the fixed duration for information was too long, so by optimizing the area of information, the efficiency of information extraction could be improved, and the duration of information fixation could be reduced. In view of the problem that novice drivers have less fixation numbers, we can consider increasing the frequency of drivers’ attention to key information through bottom-up stimulation. People are attracted by elements that are different from the surrounding environment, and combined with the problem that novice drivers search for a purpose weakly, researchers have proposed possible solutions. Referring to the focus of experienced drivers, researchers have made the key information one color and differentiated it from the background color in each driving stage. Information nontransparency is another problem existing in the current interface. With the increase of driving experience, the driver would gradually understand the influence of the traction/braking force on the speed change, but the novice drivers do not understand the correlation between the two, and make the speed as close to the authorized speed as possible through frequent traction and braking operations. However, the results are not satisfactory. Consider adding the speed prediction curve in the interface after the traction/braking force is applied, or calculate the traction/braking force that should be applied according to the current vehicle speed and authorized speed so as to provide a reference for the drivers’ driving operation.

There were some limitations in this study. Our research focused on the impact of experience on visual behavior and driving performance. The findings can support interface optimization, but the influence of other factors, such as gender, age, and personality, was not explored, although these factors were taken into account during the participant recruitment and trial design phases. If we continue to study interface optimization in depth, we need to consider the driving habits and information observation preferences of different drivers and whether they are related to factors such as age and personality so as to better analyze the advantages and disadvantages of the current interface design. This way, we can promote the development of intelligent interfaces.

## Conclusions

Experience can reduce individual differences in driving performance, increase overall operational consistency, and reduce the likelihood of hazard occurrences. Novice drivers can quickly master operation skills after a short training period, but they need to understand the correlation between the vehicle speed and traction or braking operations through a longer training, and acquire standard operating procedures and driving skills. Experience changes the visual behaviors of drivers such that their information acquisition methods gradually change from long-term and low-frequency gazes to short and high-frequency gazes and form a more targeted information search mode. Increasing the number of fixations of useful information and reducing the average fixation duration of information can help improve driving performance. Changing the area of interface information can also improve the extraction speed of key information such as the speed control area, planning area, and braking force, thus bridging the gap between novice and experienced drivers. The results can provide new ideas for high-speed train interface developers. Through interface optimization, the differences in driving performance caused by experience can be reduced, training costs can be reduced, and driving safety can be improved.

## Data Availability

All data generated or analyzed during this study are included in this published article.
